# The Role of Lattice Defects on the Optical Properties of TiO_2_ Nanotube Arrays for Synergistic Water Splitting

**DOI:** 10.1021/acsomega.3c00965

**Published:** 2023-09-01

**Authors:** Manel Machreki, Takwa Chouki, Georgi Tyuliev, Mattia Fanetti, Matjaž Valant, Denis Arčon, Matej Pregelj, Saim Emin

**Affiliations:** † Materials Research Laboratory, University of Nova Gorica, Vipavska 11c, 5270 Ajdovščina, Slovenia; ‡ Institute of Catalysis, Bulgarian Academy of Sciences, Acad. G. Bonchev St., Bldg. 11, Sofia 1113, Bulgaria; § Institute “Jožef Stefan”, Jamova 39, 1000 Ljubljana, Slovenia; ∥ Faculty of Mathematics and Physics, University of Ljubljana, Jadranska c. 19, SI-1000 Ljubljana, Slovenia

## Abstract

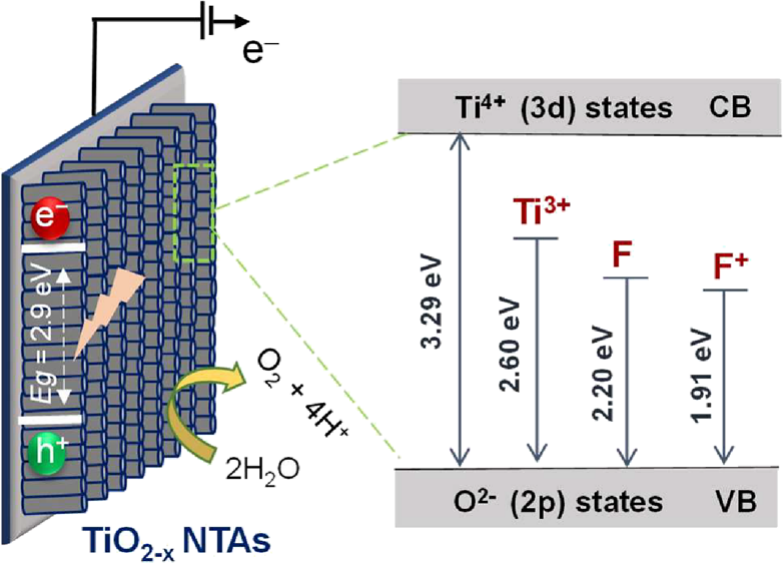

In this study, we report a facile one-step chemical method to synthesize reduced titanium dioxide (TiO_2_) nanotube arrays (NTAs) with point defects. Treatment with NaBH_4_ introduces oxygen vacancies (OVs) in the TiO_2_ lattice. Chemical analysis and optical studies indicate that the OV density can be significantly increased by changing reduction time treatment, leading to higher optical transmission of the TiO_2_ NTAs and retarded carrier recombination in the photoelectrochemical process. A cathodoluminescence (CL) study of reduced TiO_2_ (TiO_2–*x*_) NTAs revealed that OVs contribute significantly to the emission bands in the visible range. It was found that the TiO_2_ NTAs reduced for a longer duration exhibited a higher concentration of OVs. A typical CL spectrum of TiO_2_ was deconvoluted to four Gaussian components, assigned to F, F^+^, and Ti^3+^ centers. X-ray photoelectron spectroscopy measurements were used to support the change in the surface chemical bonding and electronic valence band position in TiO_2_. Electron paramagnetic resonance spectra confirmed the presence of OVs in the TiO_2–*x*_ sample. The prepared TiO_2–*x*_ NTAs show an enhanced photocurrent for water splitting due to pronounced light absorption in the visible region, enhanced electrical conductivity, and improved charge transportation.

## 1 Introduction

Titanium dioxide (TiO_2_) has been recognized as a suitable material in photocatalysis (PC), photovoltaics, photosensors, and photoelectrochemical (PEC) studies.^1^ TiO_2_ is an attractive semiconductor due to its nontoxicity, low cost, and good stability.^2^ However, the photocatalytic properties of TiO_2_ are limited by the optical absorption window, which is limited in the UV region. Tuning the light absorption (e.g., through tuning the band gap) of TiO_2_ in the visible region is the ultimate goal to prepare efficient photoactive catalysts.^3,4^ Several approaches have been introduced in the past to address limited light absorption, including the formation of heterostructures, surface photosensitization, metal deposition, and heteroatom doping.^5^ Besides these steps, oxygen vacancies (OVs) have been proven to modulate the optical properties,^6,7^ electron transport,^8^ and catalytic activities of TiO_2_.^9−11^ Especially, to design a photoactive TiO_2_ photocatalyst, it is crucial to understand the contribution of OVs in TiO_2_ to the photocatalytic reaction. Owing to their elusive diluted nature, it has been challenging to detect these defects. Spectroscopic techniques as a powerful tool are often used to detect OVs in metal oxides.^11,12^ Cathodoluminescence (CL) spectroscopy allows to elucidate the emission spectral bands caused by different transitions such as the band edge, OVs, surface and interstitial states, and color centers.^13−17^ Therefore, CL techniques were extensively used to study the emissions associated with OVs in metal oxides such as CeO_2_,^18^ Y-doped ZrO_2_,^19^ and TiO_2_.^12,20−22^ Analysis of the emission properties of the TiO_2_ sample is especially relevant since these contain information about possible lattice defects that are essential for the design of efficient photocatalysts. Mikhailov et al. have proposed that the CL spectrum of TiO_2_ particles in the 300–1200 nm range is composed of multiple bands.^23^ Ho et al. found that by altering the annealing conditions of TiO_2_, the CL band intensity can be tuned.^24,25^ Naldoni et al. demonstrated that black TiO_2_ powder samples show greater CL intensity bands compared to pristine TiO_2_ due to different radiative transitions, which are related to OV intragap states or to the self-trap exciton.^26^ To our best knowledge, there are few reports on the detailed CL properties of TiO_2_ nanotube arrays (NTAs).^27,28^ Moreover, there is a lack of systematic studies on the CL properties of defect states in reduced TiO_2_ NTAs.

The aim of this study is to show the CL behavior of TiO_2_ and its dependence on defect concentration. TiO_2_ NTAs were prepared using a one-step anodization method in ethylene glycol (EG) following a heat treatment in air at 500 °C. Reduced TiO_2_ NTAs with different degrees of OVs were synthesized utilizing a chemical approach with NaBH_4_ at various reduction times. The evolution of the intrinsic defects was investigated using electron paramagnetic resonance (EPR), cathodoluminescent (CL), X-ray photoelectron (XPS) spectroscopy, and PEC techniques. According to XPS analysis, the densities of defects can be tuned with the reduction time. The capability of the CL technique to investigate various charge states of point defects was displayed. It was demonstrated that TiO_2–*x*_ NTAs treated for longer times exhibit enhanced emission properties. As a result, the prepared TiO_2–*x*_ NTAs showed enhanced PEC water splitting due to the improved photocurrent response. This study enriches our knowledge of OVs on the catalytic activities of reduced TiO_2_ NTAs.

## 2 Experimental Section

### 2.1 Chemicals and Materials

Titanium foil (Ti, 0.2 mm thick, 99.9% purity) was obtained from Ankuro Int. GmbH (Germany). Nitric acid (HNO_3_, 65%) was purchased from Carlo Erba Reagents GmbH (Germany). Hydrofluoric acid (HF, 48–51%) was purchased from Fisher Scientific. EG (99%), hydrogen peroxide (H_2_O_2_, 99%), ammonium fluoride (NH_4_F, 99.8%), and sodium borohydride (NaBH_4_, 97%) were purchased from Alfa Aesar UK. A graphite block (99.9%) was purchased from Beijing Great Wall Co., Ltd. (China). Deionized water was used in all the experiments.

### 2.2 Preparation of TiO_2_ Nanotubes

The preparation of TiO_2_ NTAs followed an anodization process.^29−32^ First, we polished Ti foil with different abrasive papers and then cleaned it in an ultrasound bath using acetone, ethanol for 5 min, and deionized water for 10 min. The Ti foil was chemically etched in a 20 mL solution of HF and HNO_3_ (HF/HNO_3_/H_2_O = 1:4:5 in volume) for 30 s and washed with water. The anodization process was performed in a two-electrode system, using Pt as the counter electrode and Ti foil as the working electrode at 30 V for 2 h. The electrolytes were 2.5% H_2_O (2.5 mL) and 0.28 wt % NH_4_F in EG (97.5 mL). After the anodization, the film was sonicated in methanol for a short time, and then rinsed with deionized water, and finally dried with nitrogen (N_2_) gas. The obtained TiO_2_ film was annealed at 500 °C in air for 1 h (heating/cooling rates of 2 °C min^–1^). For a controlled reduction of TiO_2_, the annealed samples were dipped in 1 M NaBH_4_ solution for different times (from 0 to 24 h).

### 2.3 Sample Characterizations

X-ray diffraction (XRD) measurements of the TiO_2_ NTAs were performed by using a MiniFlex 600 W (Rigaku) diffractometer (Cu Kα radiation). Phase identification was carried out with the PDXL software. The reflectance studies were performed using a Lambda 650 UV–vis spectrophotometer (PerkinElmer). Surface morphology of the films and the CL spectra were taken using a scanning electron microscope (SEM) JSM 7100F (JEOL) equipped with a field-emission electron gun. The electron-stimulated luminescence emitted by the sample was collected and concentrated toward a spectrometer with a 3 nm spectral resolution using a CL apparatus (Gatan MonoCL4) installed on the same SEM. The CL measurements of all samples were performed under identical experimental conditions. The SEM acceleration voltage and beam current were set to 20 kV and 3.3 nA for all analyses. The CL spectra were taken in the wavelength range from 300 to 750 nm. They were corrected by grating response and detector response in the 350–750 nm wavelength range. Transmission electron microscopy (TEM) studies were performed with a JEOL 2100F operated at 200 kV. The ex situ XPS measurements were performed using an electron spectrometer ESCALAB MkII (VG Scientific) operating at a base pressure of ∼5 × 10^–8^ Pa. The “as- prepared” samples were attached on copper sample holders with conductive adhesive tape. C 1s, O 1s, Ti 2p, Ti LMM, and VB regions were recorded with Al Kα (*h*ν = 1486.6 eV) radiation at a total instrumental resolution of ∼1.1 eV. All spectra were calibrated by using the C 1s peak at 285.0 eV as a reference. The surface composition was evaluated from the photoelectron intensities divided by the corresponding photoionization cross sections taken from Scofield.^33^ To confirm the presence of Ti^3+^ and OVs in the sample, the EPR was recorded on a home-built X-band EPR spectrometer equipped with a Varian E101 microwave bridge and a Varian TEM104 dual-cavity resonator. Physisorption isotherms were recorded by using N_2_ adsorption–desorption at 77 K (Autosorb iQ-XR from Quantachrome/Anton Paar). The specific surface area and pore characteristics were estimated using the Brunauer–Emmett–Teller (BET) method and the Barrett–Joyner–Halenda (BJH) method. PEC measurements were done in a three-electrode system using a so-called cappuccino cell (EPFL, Switzerland). An O-ring was used to define the electrode area at 0.283 cm^2^. The potential of the working electrode were controlled by a potentiostat (EDAQ SP1). A graphite plate and Hg/Hg_2_SO_4_ electrode were used as the counter and reference electrodes, respectively. The electrochemical impedance spectroscopy (EIS) of TiO_2_ NTAs was studied in a 0.1 M Na_2_SO_4_ solution. The applied frequency ranged from 1 Hz to 100 kHz with an amplitude of 5 mV. The measured EIS data were fitted using the ZView software. The Mott–Schottky (M–S) plots were obtained from impedance potential plots performed in the dark at different frequencies.

## 3 Results and Discussion

### 3.1 Structural Characterization

**Figure 1:**
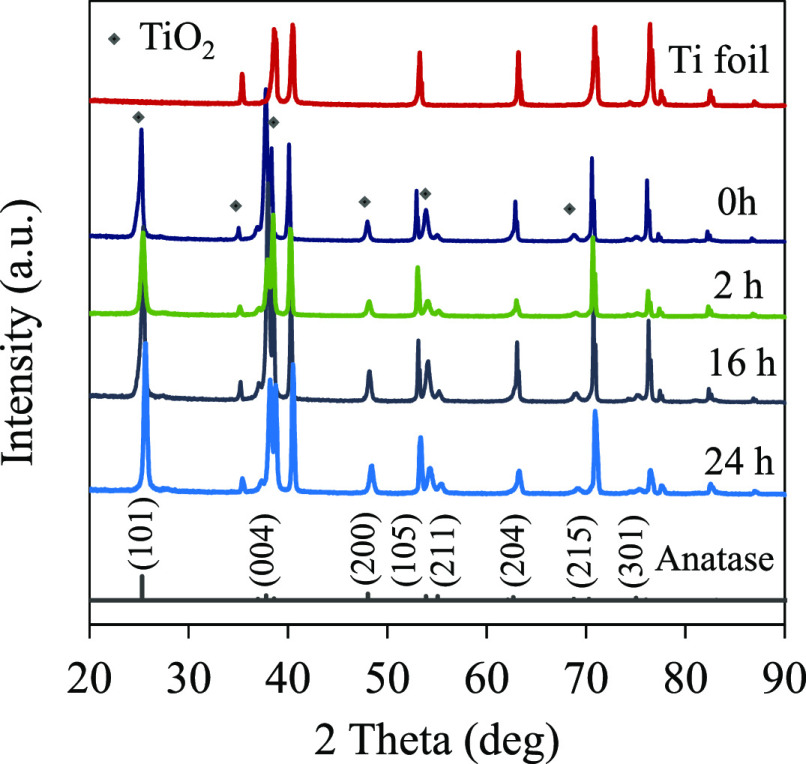
XRD of the TiO_2_ and TiO_2–*x*_ nanotubes obtained in a 1 M NaBH_4_ solution at different times. With asterisk are given the TiO_2_ peaks.

After the anodization procedure, TiO_2_ NTAs were crystallized at 500 °C. XRD patterns of the pristine TiO_2_ (0 h) and TiO_2–*x*_ NTAs prepared at different NaBH_4_ reduction treatment times are shown in Figure 1.^34^ The intense diffraction peaks at 25.28, 37.80, 48.05, 53.89, 62.30, and 75.03° correspond to (101), (004), (200), (105), (204), and (215) crystallographic planes of the anatase phase of TiO_2_ (I41 (141); PDF no. 9008213). The XRD patterns of TiO_2–*x*_ NTAs were almost identical to the pristine TiO_2_. These results suggest that the crystal structure was unchanged during the chemical treatment.^35^ A peak shift toward a higher 2θ degree (a low crystal lattice spacing) can be observed for treated samples. The phenomenon implies the condensed crystal lattice due to the removal of oxygen from the TiO_2_ lattice and the partial replacement of Ti^4+^ by Ti^3+^.^10^ In early studies, Sun et al. have reported a facile route to enhance electrical conductivity and improve the electrochemical behavior of NTA electrodes by controlling the formation of substoichiometric titanium oxides (Ti_*n*_O_2*n–*1_) known as Magnéli phases.^11^ The authors have produced Magnéli phases by heat treatment at 450 °C in a N_2_ atmosphere. In our study, the chemical reduction disfavors the formation of crystalline substoichiometric TiO_2_ phases since there are missing diffractions typical for Ti_4_O_7_, Ti_5_O_9_, Ti_6_O_9_, etc. (Figure S1).

**Figure 2:**
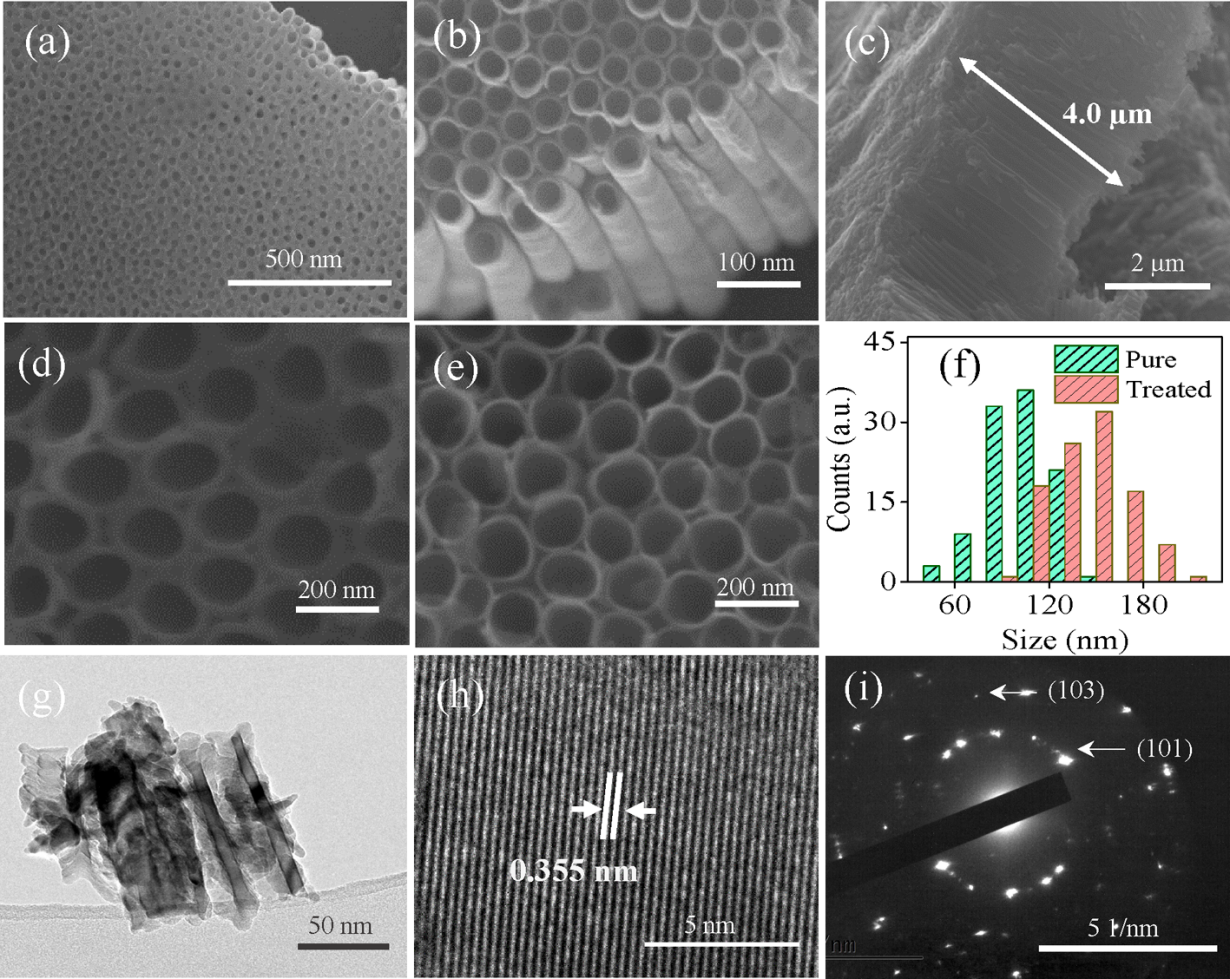
(a, b) SEM images of TiO_2_ NTAs at different magnifications. (c) Cross-section of the TiO_2_ NTAs. SEM images of (d) TiO_2_ and (e) TiO_2-x_ NTAs. (f) Corresponding size distribution diagram of the inner diameter of TiO_2_ (d) and TiO_2-x_ NTAs (e). (g) TEM image of TiO_2_ tubes. (h) HR-TEM image of TiO_2-x_ (treated for 24 h) lattice and (i) SAED pattern taken from (g).

Figure 2 shows SEM images of TiO_2_ samples before and after NaBH_4_ treatment. It is obvious that the pristine TiO_2_ NTAs exhibit a uniform morphology with a nanotubular structure, as revealed in Figure 2a,b. The film thickness of the TiO_2–*x*_ NTs is estimated to be on the order of 4.0 μm (Figure 2c). The morphology of the TiO_2–*x*_ film resembles that of the pristine one. It is obvious that NaBH_4_ treatment maintains the nanotubular structure of the TiO_2–*x*_ NTAs. However, closer observation reveals that the walls of reduced TiO_2–*x*_ NTAs become thinner (Figure 2d,e). This indicates that NaBH_4_ indeed reacted with TiO_2_. The average inner diameter of TiO_2–*x*_ NTAs is in the 120 to 190 nm range and is larger than that of pristine TiO_2_ (Figure 2f). According to a previous report, thinner walls benefit the charge transport in TiO_2_ NTAs.^34^

Figure 2g shows a TEM image of the broken TiO_2_ nanotubes. High-resolution TEM reveals that the crystal plane spacing is equal to 0.355 nm, corresponding to the (101) crystal plane, which shows that the TiO_2_ NTAs maintain good crystallinity (Figure 2h). The selected area electron diffraction (SAED) pattern indicates that these tubes are polycrystalline (Figure 2i).^36^ The diffraction rings in the SAED can be indexed to the (101) and (103) of TiO_2_ NTAs. The TEM image also shows an amorphous layer in some parts of the TiO_2–*x*_ nanotubes (16 h), which are mainly located at the edges or close to the tube walls (Figure S2). However, the sample that was treated for 24 h did not follow this exact trend. In this instance, the amorphous layer’s thickness is increasing primarily at certain spots (Figure S2). This change in crystallinity in the outer layer of TiO_2–*x*_ nanotubes seems to occur mostly as a result of the partial dissolution and reformation of TiO_6_^2–^ octahedrons caused by NaBH_4_ in water solution.^35^

### 3.2 Optical Absorption Studies

**Figure 3:**
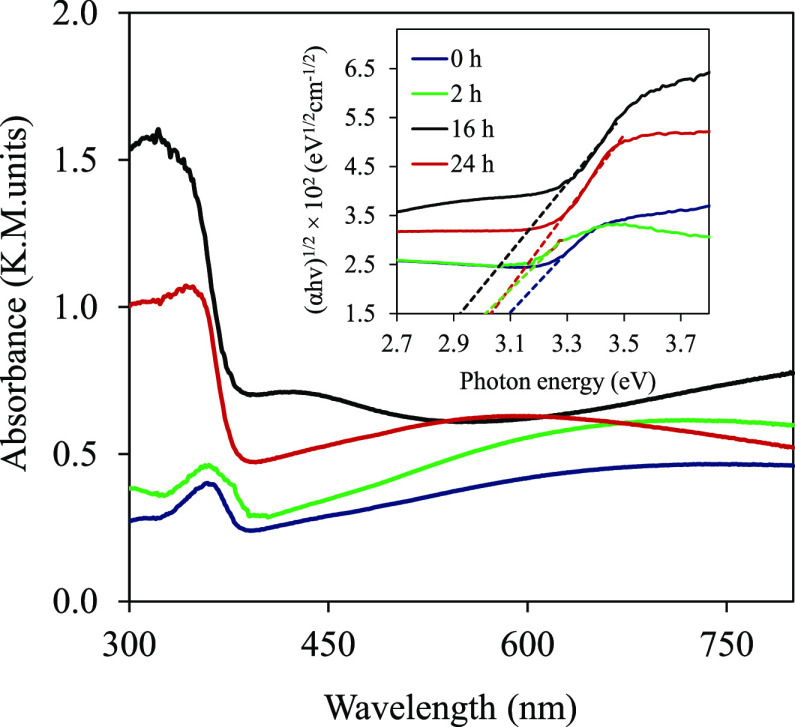
Absorbance spectra of TiO_2_ and TiO_2–*x*_ NTAs (in 1 M NaBH_4_ solution for 2, 16, and 24 h). The inset shows the Tauc plot.

To qualitatively describe the presence of OVs in TiO_2–*x*_ NTA films, absorbance spectra were recorded (Figure 3). For the pristine TiO_2_ NTAs, a sharp absorption edge around 380 nm corresponds to the band edge of anatase TiO_2_. The TiO_2–*x*_ NTAs show a stronger light absorption than the pristine ones over the entire ultraviolet–visible region. Moreover, TiO_2–*x*_ NTAs showed an additional absorption peak extending from 400 nm to the near-infrared range. The samples treated for 16 h showed the greatest change in light absorption. Clearly, the distinct differences in optical absorption behavior of TiO_2_ and TiO_2–*x*_ samples may be due to the presence of vacancies (Ti^3+^ sites formed by chemical reduction treatment), which result as trap states.^37^ The band gaps (*E*_g_) of TiO_2_ and TiO_2–*x*_ NTA films were calculated (assuming indirect transition) from the Tauc plots:^38^ (α*h*v)^1/*n*^ = *A* (*h*v – *E*_g_), where α represents the absorption coefficient, *h*v is the incident photon energy, and *A* is the proportionality constant.^38^ Depending on the reduction times of the 0, 2, 16, and 24 h films, the *E*_g_ values of TiO_2_ samples are determined to be 3.10, 3.03, 2.92, and 3.01 eV, respectively (inset Figure 3). Therefore, all TiO_2–*x*_ NTAs show slightly narrower *E*_g_ in comparison with pristine TiO_2_, which is consistent with earlier studies.^34^ A red shift of absorption edges and narrowed band gaps of TiO_2–*x*_ are associated with the OV levels.^39^ It is noticed that the decrease of *E*_g_ in TiO_2–*x*_ NTAs reaches saturation after a certain time (16 h).^40^ It was found that the strong light absorption of the TiO_2–*x*_ NTAs benefits their PC performance.^41^

### 3.3 XPS and EPR Studies

The XPS measurements were performed to study the change in surface chemical bonding of TiO_2_ NTAs including the OV content and valence band (VB) position. In both pristine and reduced TiO_2_ samples, the binding energies of Ti 2p_1/2_ and Ti 2p_3/2_ are about 464.2 and 459.1 eV, respectively, which are a hallmark of the Ti^4+^ – O bonds (Figure S3a).^42^ The Ti 2p core level spectra of treated and untreated samples show an insignificant difference, as shown in Figure S3b,c. A possibility preventing us to detect the Ti^3+^ centers could be that, at the surface, low-coordinated titanium atoms are easily reacted with oxygen or other species in the atmosphere and only those atoms, which are deeper in the film, and therefore not detected by XPS, retain their lower oxidation state.^43,34^

**Figure 4:**
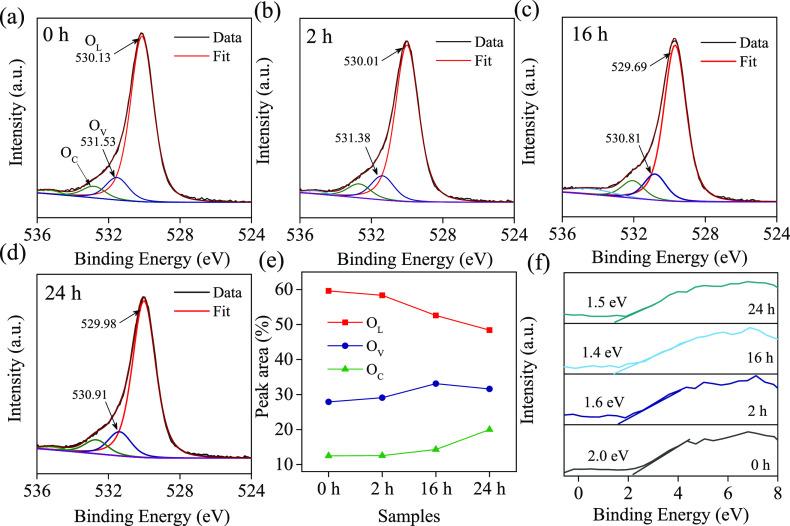
(a–d) Deconvoluted O 1s XPS spectra of TiO_2_ NTAs at different treatment times, where each spectrum is fitted with three components: chemisorbed oxygen (O_C_), lattice oxygen (O_L_), and oxygen vacancies (O_V_). The peak positions (in eV) for O_L_ and O_V_ as resulting from the fits are provided. (e) The percentage of area under the deconvoluted peaks (O_L_, O_V_, and O_C_) as a function of sample type. (f) XPS valence band edges of the TiO_2_ and TiO_2–*x*_ samples.

XPS spectra for O 1s are recorded and fitted with three Gaussian curves, which are attributed to lattice oxygen (O_L_), O_V_, and chemisorbed oxygen species (O_C_), respectively (Figure 4a–d).^44^ The O_L_ peak at low binding energies comes from the lattice oxygen atoms in a fully coordinated TiO_2_ with the Ti^4+^ ions mainly on the surface. The O_V_ peak at medium binding energies is associated with the presence of OVs (i.e., with hydroxyl species due to the adsorption of moisture at OVs) at the TiO_2_ surface,^45^ and the O_C_ peak at high binding energy corresponds to the loosely adsorbed species, dissociated oxygen, or other species, such as O_2_, H_2_O, and C–O, at the surface of TiO_2_.^46^Figure 4e demonstrates that surface O_V_ concentrations gradually increase with the chemical reduction time.^47^ While the O_C_ peaks are improved throughout the reduction time, this implies that the amount of loose chemisorbed oxygen species on the surface increases.^44^Figure 4f illustrates the VB spectra of TiO_2_ NTAs at various reduction times. The VB is determined at the XPS band edge, which is positioned at 2.0, 1.6, 1.4, and 1.5 eV and corresponds to 0, 2, 16, and 24 h, respectively. This result suggests that chemical reduction treatment caused a shift in the VB position of TiO_2_ NTAs’ surface. It also demonstrates that the NaBH_4_ treatment introduced Ti^3+^/OVs into TiO_2–*x*_, probably due to a localized-state-induced shift in the VB position.^34^ The energy diagram of TiO_2_ and TiO_2–*x*_ NTAs was constructed by using the VB values and the optical band gap obtained from the absorption spectroscopy (Figure S4).

Furthermore, EPR measurements were carried out to investigate the unpaired electrons of defect-associated centers and vacancies at low temperatures.^34^ As reported in other studies, the EPR signals of Ti^3+^ defects, OVs, and O^2–^ are located at *g* values of 1.960–1.990, 2.000–2.016, and 2.020, respectively.^10,48,49^ As shown in Figure S5, the treated sample TiO_2_ at 16 h gives a weak peak with a *g* value of 1.985, which is attributed to the existence of paramagnetic Ti^3+^ species, while the intensive signal with a *g* value of 2.004 is assigned to OVs.^50^ The signal of metastable surface Ti^3+^ species is in general low, possibly due to the easy oxidization of Ti^3+^ centers when exposed to air (e.g., due to reaction with atmospheric O_2_, H_2_O, etc.).^48^ The signal intensity of the *g* = 2.004 EPR signal is apparently higher, which suggests that the NaBH_4_ chemical treatment creates OVs deeper in the TiO_2_ lattice.^51^ In our EPR measurements, a signal at a *g*value of 2.02 assigned to superoxide radical anions was also detected. A possible mechanism for the creation of superoxide radical anions includes the presence of the surface Ti^3+^, which tends to adsorb and reduce atmospheric O_2_ to O^2–^.^48^ Finally, the EPR spectra show that the EPR signal intensity of the TiO_2–*x*_ samples is higher than that of the parent TiO_2_. This implies the presence of OVs in the TiO_2–*x*_ sample and thus corroborates the XPS results.

### 3.4 CL Study

CL spectroscopy is a unique technique that has been used to study defects in metal oxides such as TiO_2_.^25,52,53^ CL spectroscopy measurements at low electron voltages were carried out to better quantify the impact of chemical reduction time on TiO_2_ OV defects in the “bulk” of the film.

**Figure 5:**
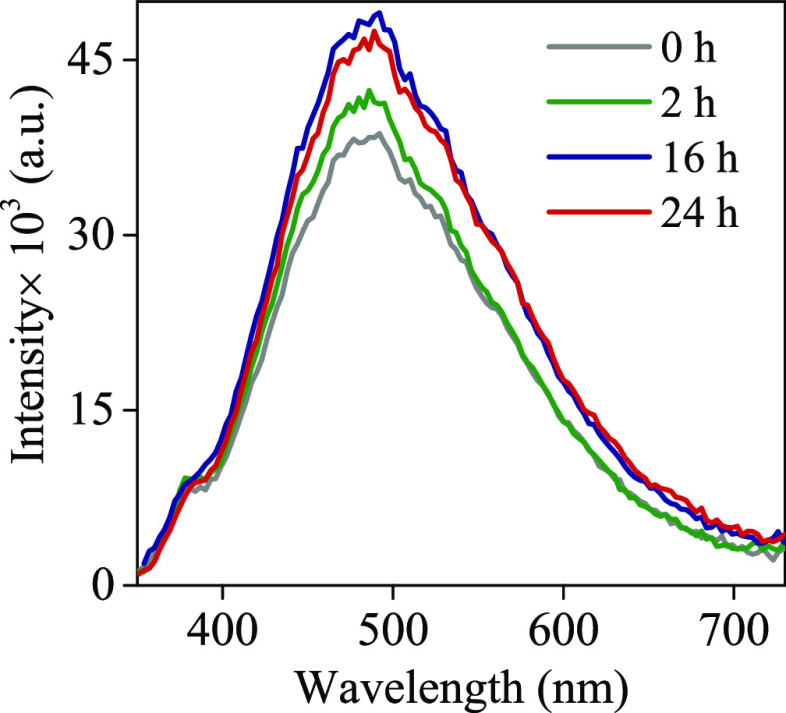
CL spectra collected for TiO_2_ (0 h) and TiO_2–*x*_ nanotubes obtained in a 1 M NaBH_4_ solution at different times.

**Figure 6:**
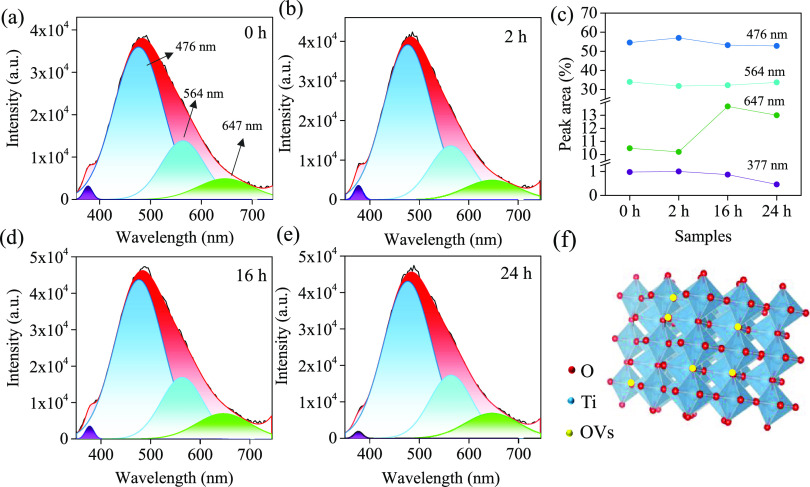
Gaussian deconvolution of the CL spectra collected from (a) TiO_2_ (0 h) and TiO_2–*x*_ nanotubes obtained by treatment in 1 M NaBH_4_ solution at different times (b) 2, (d) 16, and (e) 24 h. (c) The percentage of areas under the deconvoluted peaks as a function of four samples. (f) Schematic of the TiO_2–*x*_ crystal with OVs (red balls are O, blue balls are Ti, and yellow balls are OVs).

Figure 5 presents the CL spectra of pristine TiO_2_ and TiO_2–*x*_ NTAs reduced at different times. Compared with pristine TiO_2_, the intensity of TiO_2–*x*_ CL peaks increases when the reduction time increases. These variations in TiO_2–*x*_ CL intensity may indicate an increase in OV concentrations. It is well known that OVs can appear in a variety of charged states: (i) single ionized (*Vo*^•^), (ii) double ionized (*Vo*^••^), and (iii) neutral state (*Vo*).^54^ As a result of the OV arrangement, energy levels form within the band gap, which in principle contribute to different luminescence bands. Silva et al. reported a correlation between structural and electronic order–disorder effects in nanocrystalline TiO_2_ and the corresponding luminescence properties.^55^ In this work, the CL effects were utilized to evaluate the possible transitions associated with OVs. The CL spectra were fitted by adopting the Gaussian distribution, as displayed in Figure 6. After deconvolution of the broad CL, four components are visible in the spectra: 377 (3.29 eV), 476 (2.60 eV), 564 (2.20 eV), and 647 nm (1.91 eV). The emission band at 377 nm has been attributed to an electron band-to-band transition from the TiO_2_ CB minimum to the VB and is relatively weak.^56−58^

The remaining three emission bands in the visible range are mostly associated with shallow states or deep trap centers related to oxygen defects.^1^ The blue emission peak at 476 nm (2.60 eV) is frequently observed in TiO_2_ nanostructures and is ascribed to Ti^3+^ states and singly ionized OVs.^59−61^ In early studies, Tariq et al.^54^ identified a PL band at around 2.67 eV in rutile TiO_2_ (110) single crystals after a strong photoexcitation. Fernandez et al.^62^ reported that a blue luminescence CL peak at 2.75 eV becomes the dominant emission in the visible region after treatment in oxygen, suggesting that the de-excitation process is assisted by a shallow trap associated with an OV.^63−65^ The other band appearing at 564 nm (2.20 eV) is due to de-excitation from lower levels in the OVs of the TiO_2_ lattice to the ground state.^57,66^ Yang et al. assigned the PL bands at 2.21 and 2.39 eV to the OVs on the surface of TiO_2_ NTAs.^67^

The electron pair trapped in the vacancy cavity (*V*_O_) will form the so-called F center due to the loss of one oxygen atom in the TiO_2_ lattice.^68^ As described in eqs 1 – 4, an electron (e^–^) in the F center may occupy the nearby Ti^4+^ ion, resulting in a Ti^3+^ center and a F^+^ center, which will create shallow and deep trap states as given below:
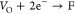
1
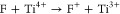
2
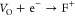
3
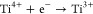
4

The sub-band located at 476 nm can be attributed to Ti^3+^ states, whereas CL emission centered at 564 nm originates from the shallow trap state linked with the F center.^57,67,69,70^ Finally, the red emission peak at 647 nm (1.91 eV) was attributed to the oxygen interstitial of the color center of TiO_2_.^22,71^ It may be corresponded to electron transition from the F^+^ center to the acceptor level just above the VB.^61,68,72^

**Figure 7:**
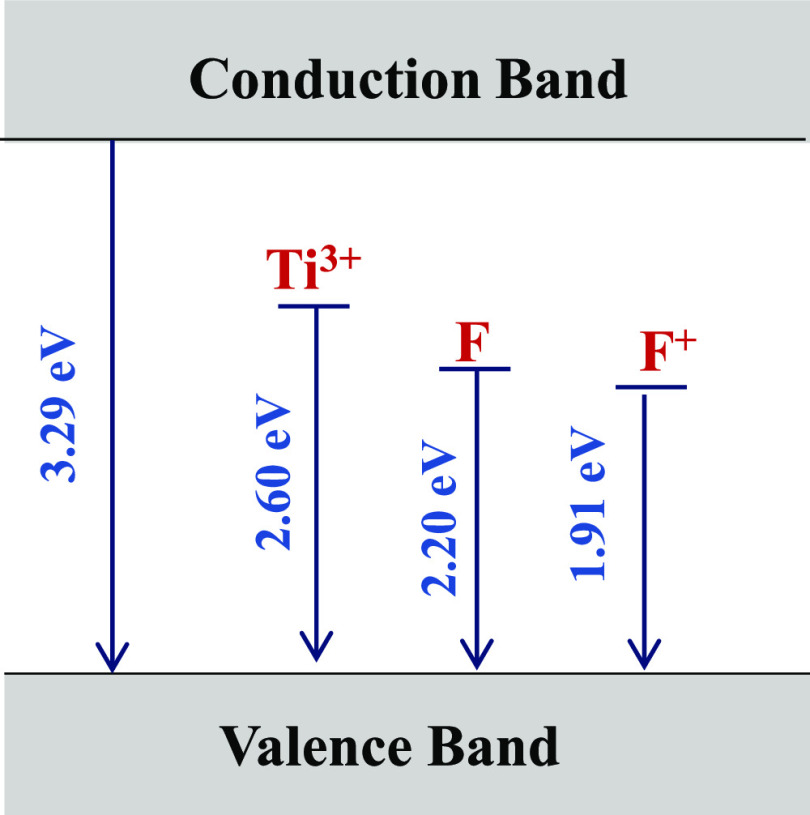
Illustration of the structural model of energy states within the band gap of TiO_2_ NTAs, corresponding CL peaks.

The intensity ratio (area ratio) of the emission band at 647 nm in the TiO_2–*x*_ samples shows an increase following the reduction time compared to other bands (Figure 6c). This indicates that the density of OV sites in the “bulk” film is enhanced after reduction treatment at longer durations.^26,61^ Furthermore, we propose a model for the energy levels within the band gap of TiO_2_ NTAs by combining the deconvoluted CL peaks and compared with previous reports,^1,57^ as illustrated in Figure 7.

### 3.5 Adsorption–Desorption Isotherms of N_2_

**Figure 8:**
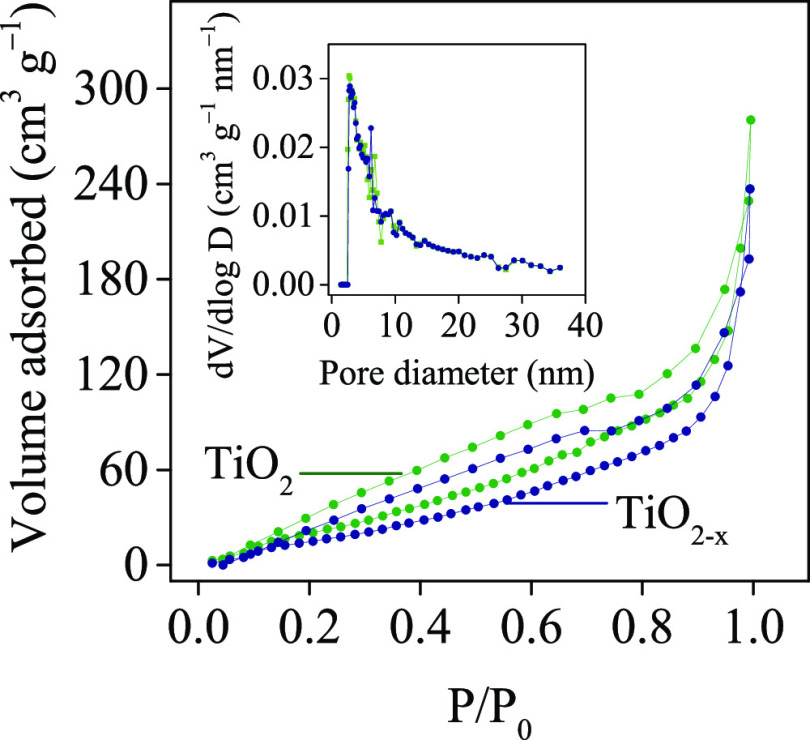
Adsorption–desorption isotherms of N_2_ on TiO_2_ and TiO_2–*x*_ NTAs (16 h). The inset shows the corresponding pore size distribution curves.

To evaluate the specific surface area of NTAs, N_2_ gas adsorption–desorption isotherms of TiO_2_ and TiO_2–*x*_ (16 h) were recorded. As shown in Figure 8, each sample exhibits a type IV isotherm with a hysteresis loop typical for mesoporous materials.^73,74^ The BET surface areas of TiO_2_ and TiO_2–*x*_ NTAs are 89 and 73 m^2^ g^–1^, respectively. Thus, the TiO_2–*x*_ NTAs owns a small surface area than the pristine sample, which should be due to the ultrafine geometric characteristic of the nanotubes fabricated by the modified rapid anodization method.^74^ The inset in Figure 8 shows a narrow pore size distribution for both samples under 5 nm. The mesoporous size distribution of the TiO_2_ and TiO_2–*x*_ NTAs were not greatly different, indicating that the pore size uniformity of the two materials was near similar. The calculated pore sizes from the isotherms are smaller than those seen in the SEM images for the titania tube (Figure 2). These mesopores could be attributed to the empty space left between the tubes (outer walls). It seems that the NaBH_4_ treatment does not significantly modify the pore size distribution of TiO_2_ NTAs.^75^ Moreover, the obtained specific surface area values are close to those recorded in previous studies with this difference that tube diameter and thickness slightly differ.^76−80^

### 3.6 PEC Studies

**Figure 9:**
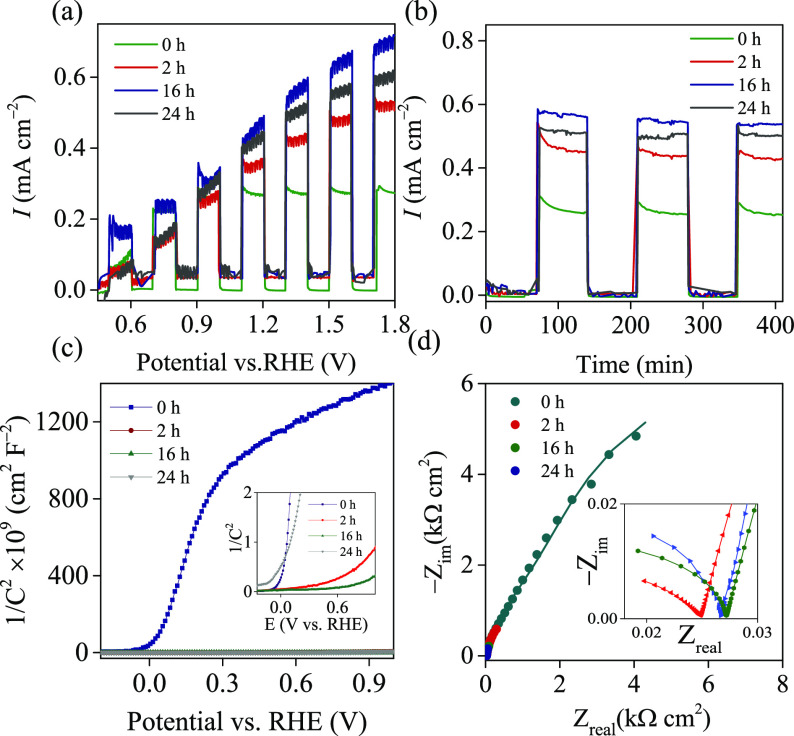
Comparison of TiO_2_ and TiO_2–*x*_ NTAs at different reduction times: (a) LSV in 0.1 M Na_2_SO_4_ (pH 5) recorded at 5 mV/s under chopped illumination (λ 370 nm). (b) Current vs time recorded at 1.5 V vs RHE. (c) M–S plots of TiO_2_ and TiO_2–*x*_ NTA electrodes at 5 kHz. (d) Nyquist plot of TiO_2_ and TiO_2–*x*_ NTAs at 1.23 V vs RHE under illumination (the inset shows enlargement of Nyquist plots for the TiO_2–*x*_ NTAs).

We studied the photocurrents of TiO_2–*x*_ NTA photoanodes as a function of NaBH_4_ treatment time. Figure 9a shows that the TiO_2–*x*_ NTAs exhibit higher photoactivity for water oxidation than the pristine TiO_2_ NTAs (Figure S6). In the present study, the photocurrent density of the film (mA cm^–2^) was determined based on its geometric area. A threefold increase in photocurrent density is seen for the 16 h NaBH_4_-treated sample. The photocurrent gradually increases with the applied positive potential, indicating that the electric field enhances the charge separation. At higher potentials, a saturation of the photocurrent is observed in all samples. It is known that for NTAs, the space charge layer is generated at the tube walls and deviation from the classical Gartner model for the potential dependence of the photocurrent is observed. PEC reactions occur primarily on the catalyst surface.^34^ The reaction efficiency and associated mechanisms would be strongly influenced by surface properties, such as surface OV. The density of electron trap states can be adjusted by creating OVs on the catalyst’s surface.^81^ When the surface interfacial electron transfer rate and photocurrent are large, the PEC performance of TiO_2–*x*_ is high. According to the literature, electron–hole recombinations are inhibited when the OVs act as charge carrier traps and adsorption sites.^34^ Longer NaBH_4_ treatment, 24 h, causes a high degree of OVs in the TiO_2_ NTAs, which gives reduced PEC efficiency. Similar results for water oxidation are reported on fluorinated TiO_2_ nanoporous films.^82^ Longer NaBH_4_ treatment, 24 h, causes a high level of OVs (33.1%) in the TiO_2–*x*_ NTAs compared to 16 h (31.6%), which gives reduced PEC efficiency..^82^ Corby et al.^83^ reported the performance of nanostructured WO_3_ films with different concentrations of bulk OVs and found that the medium OV concentration gave the maximum photocurrent. High levels of defects result in a greater probability of trap-mediated recombinations. The holes that are trapped deeply in the material are unable to contribute to water oxidation, and thus higher incidences of trap-assisted recombination result in lower photocurrents.^83,84^ The photocurrents of TiO_2_ and TiO_2–*x*_ NTA photoanodes as a function of NaBH_4_ treatment time were also studied in the presence and absence of a H_2_O_2_ hole scavenger (Figure S7). An increase in the photocurrent has been observed due to the efficient charge injection from the scavenger to the photoanode. As a general trend, there is an increase in the photocurrent in all studied samples. Figure 9b presents the chronoamperometry test (*I–t*) of the photo responses of TiO_2–*x*_ and TiO_2_ NTAs under chopped illuminations at 1.5 V vs reversible hydrogen evolution (RHE). The treated and untreated samples exhibited good stability. Under dark conditions, the lack of photocurrent shows that the film is not catalytically active while TiO_2–*x*_ NTAs treated for 16 h reported the highest photocurrent value of 0.6 mA cm^–2^ at 1.5 V vs RHE.

The PEC performance of TiO_2_ NTAs was further characterized by using M–S plots (Figure 9c). Strong dependence between the capacitance and applied potential was observed for the TiO_2_ NTA electrode, which indicated that the capacitance was controlled by the space charge layer. Due to its expected higher conductivity, the TiO_2–*x*_ NTA electrode showed only a weak correlation between capacitance and applied potential (e.g., metallic characteristics).^36^ An important parameter of the semiconductor electrode is its flat-band potential (*V*_FB_). According to the depletion layer model, the capacitance of the semiconductor space charge layer (*C*) depends on the applied potential (*V*) and can be estimated from the M–S equation:^38^
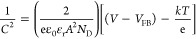
5where e is the electron charge, ε_0_ is the vacuum permittivity, ε_r_ represents the relative permittivity (dielectric constant) of TiO_2_ (ε_r_ = 10),^34^*A* is the surface area of the electrode, *k* is the Boltzmann constant, *N*_D_ is the donor density, and *T* is the absolute temperature. Figure S8 and Table S1 present the results of the flat-band potential for different samples at different frequencies. Clearly, the NaBH_4_ reduction treatment time alters the flat-band potential position. Negative *V*_FB_ values for the TiO_2–*x*_ NTAs might be attributed to the presence of Ti^3+^ affecting the surface properties of TiO_2_, which in turn may enhance the electrical conductivity of the TiO_2–*x*_ NTAs.^34,82^ The negative shift of *V*_FB_ assumes that the TiO_2–*x*_ NTA electrode could exhibit improved PEC activity.^83^ The donor density (*N*_D_) of the pristine and TiO_2–*x*_ NTAs was determined from the slope of the M–S plots. Importantly, the TiO_2–*x*_ NTAs show substantially smaller slopes of the M–S plot compared to the pristine TiO_2_ sample, suggesting an increase in donor densities. *N*_D_ was calculated from the slopes of M–S plots using the following equation:
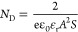
6

The *N*_D_ of the pristine TiO_2_ NTAs is 1.12 × 10^20^ cm^–3^, and the TiO_2–*x*_ NTAs for 2, 16, and 24 h are 5.40 × 10^21^, 2.51 × 10^22^, and 1.22 × 10^22^ cm^–3^, respectively. NaBH_4_ treatment increases the *N*_D_ of TiO_2_ NTAs, by creating surface OVs that serve as electron donors.^84^ The increase in *N*_D_ often enhances the electrical conductivity as well as charge transport in TiO_2_.^85^ Using the *V_FB_* and the optical band gap values it was calculated the VB and CB energy positions. It has been reported that the CB potential (*E*_CB_) of *n*-type semiconductors is 0.1 to 0.2 V more negative than their *V*_FB_ values.^38^ The energy diagram of TiO_2–*x*_ NTA samples is constructed using the measured *V*_FB_ values at 5 kHz (Figure. S9).

An alternative method of determining the *V*_FB_ is based on the measurement of the photocurrent as a function of applied potential, following the Gartner–Butler (G–B) model (Figure S10).^85^ The *V*_FB_ was estimated by the intersection of the photocurrent squared and the potential axis. The G–B equation relates the net measured photocurrent (*I*_photo_) to the extent of band bending in the semiconductor:^86^
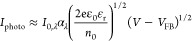
7where *I*_0,λ_ is the incident photon flux, α_λ_ (wavelength-dependent) is the material absorption coefficient, and *n*_o_ is the donor concentration (*n*_o_*≈ N*_D_). The *V*_FB_ potentials determined through G–B analysis were also found to have a negative shift with NaBH_4_ reduction treatment time. As shown in Table S2, there is no significant difference in the relative positions of *V*_FB_ obtained by Mott–Schottky or by the Butler–Gärtner model.

EIS measurements were used to evaluate the charge-transfer resistances of TiO_2_ NTAs and recombined processes at the semiconductor–electrolyte interfaces.^87,88^ A smaller arc in a Nyquist plot correlates with a smaller charge-transfer resistance on the surface of TiO_2–*x*_ electrodes.^10^ The smaller arcs, higher or lower frequencies, of the Nyquist plots decreased in the following order: 0 h > 2 h > 24 h > 16 h (Figure 9d). This result suggests that the electron transfer in the samples treated at 16 h is more efficient than that for the other samples.^34,89^

## 4 Conclusions

To summarize, TiO_2_ NTAs were synthesized using a one-step anodization method and following heat treatment in air. Through a simple one-step chemical method, reduced TiO_2–*x*_ NTAs were prepared. CL and XPS studies demonstrated that the chemical reduction time affects the concentration of OVs in the TiO_2_ lattice. The formed OVs TiO_2–*x*_ favor light absorption in the VIS range. To study the defect-state emissions in the TiO_2_ NTAs, CL spectroscopy was used. CL emission of pristine and reduced TiO_2_ films in the VIS range shows that the OV density increases with chemical reduction time. The CL spectra components can be assigned to OVs including Ti^3+^, F, and F^+^ centers. We took advantage of CL spectroscopy to resolve the contribution of OVs to the optical activity in the red region of the emission spectrum, thus providing an analytical tool for qualitative evaluations of reduced TiO_2_ films. PEC studies show that TiO_2–*x*_ NTAs improved catalytic activity for water oxidation perhaps due to a combination of factors such as light absorption and lower resistivity. The methodology for characterization of the TiO_2_ catalyst can be useful guide on how to design other metal oxide catalysts for efficient PEC energy conversion processes.

## Supporting Information Available

patterns from Magnéli phase Ti_*n*_O_2*n*–1_; high-resolution TEM images; high-resolution XPS spectra of Ti 2p at different NaBH_4_ treatment times, and deconvoluted Ti 2p core level spectra of pure and TiO_2–*x*_ films; the schematic illustration of the band positions for the pristine TiO_2_ and treated TiO_2–*x*_ at different reduction times; EPR spectra for TiO_2_ and TiO_2–*x*_ NTAs measured at 50 K; measured photocurrent density of the reduced TiO_2_ NTAs at 1.7 vs. RHE as a function of NaBH_4_treatment time in 0.1M Na_2_SO_4_ (pH 5) recorded at 5 mV/s; LSV in 0.1 M Na_2_SO_4_ with and without 0.1 M H_2_O_2_ recorded at 5 mV/s under light; Mott–Schottky plots at different frequencies; flat-band potential values for selected frequency for TiO_2_ and TiO_2–*x*_ samples; the schematic illustration of the band positions for the pristine TiO_2_ and treated TiO_2–*x*_ at different reduction times; LSV in 0.1 M Na_2_SO_4_ (pH 5) recorded at 5 mV/s of TiO_2_ and TiO_2–*x*_ NTAs at different reduction times; summary of PEC properties for TiO_2_ and TiO_2–*x*_ samples (PDF)
