# Supplementary material for: The Role of Lattice Defects on the Optical Properties of TiO2 Nanotube Arrays for Synergistic Water Splitting
Source: ACS Omega. 2023 Sep 1;8(37):33255–65. doi: 10.1021/acsomega.3c00965 (PMC10515401; doi:10.1021/acsomega.3c00965)
Supplement: Supplementary file 1 — ao3c00965_si_001.pdf [file ao3c00965_si_001.pdf]

Supporting Information

# The role of lattice defects on the optical properties of $TiO_2$ nanotube arrays for synergistic water splitting

Manel Machreki,[1] Takwa Chouki,[1] Georgi Tyuliev,[2] Mattia Fanetti,[1] Matjaž Valant,[1] Denis Arčon,[3,4] Matej Pregelj,[3] and Saim Emin[1,*]

[1]Materials Research Laboratory, University of Nova Gorica, Vipavska 11c, 5270, Ajdovščina, Slovenia

[2] Institute of Catalysis, Bulgarian Academy of Sciences, Acad. G. Bonchev St., Bldg. 11, Sofia 1113, Bulgaria

[3] Jozef Stefan Institute, Jamova c. 39, SI-1000 Ljubljana, Slovenia

[4] Faculty of mathematics and physics, University of Ljubljana, Jadranska c. 19, SI-1000 Ljubljana, Slovenia

*Corresponding author. E-mail address: saim.emin@ung.si

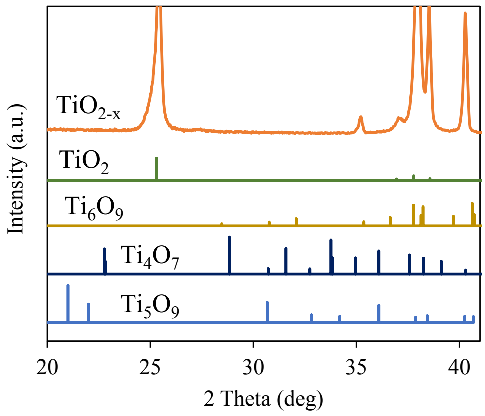


**Figure S1.** XRD of the $TiO_{2-x}$ nanotubes obtained in 1M $NaBH_4$ solution at different times with the reported XRD patterns from Magnéli phase $Ti_nO_{2n-1}$.

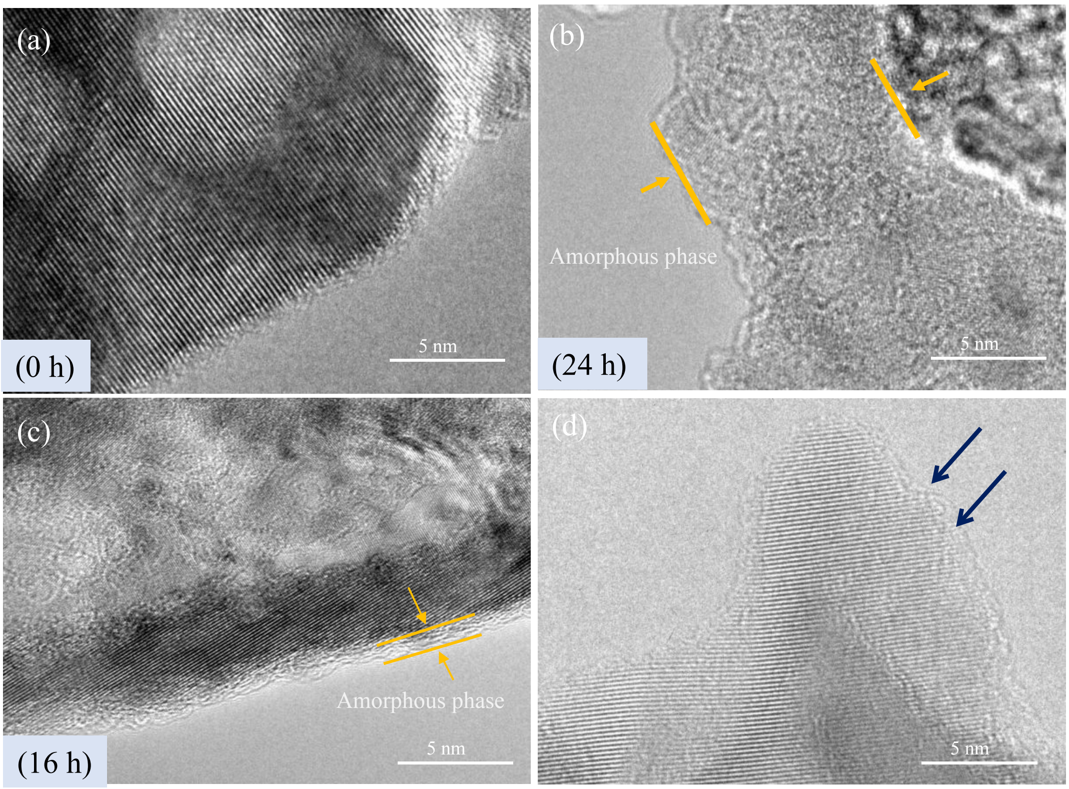


**Figure S2.** High resolution TEM images of (a) $TiO_2$ and $TiO_{2-x}$ NTAs for (b) 24 h and (c, d) 16 h. The arrows show a thin layer that is composed of amorphous phases.

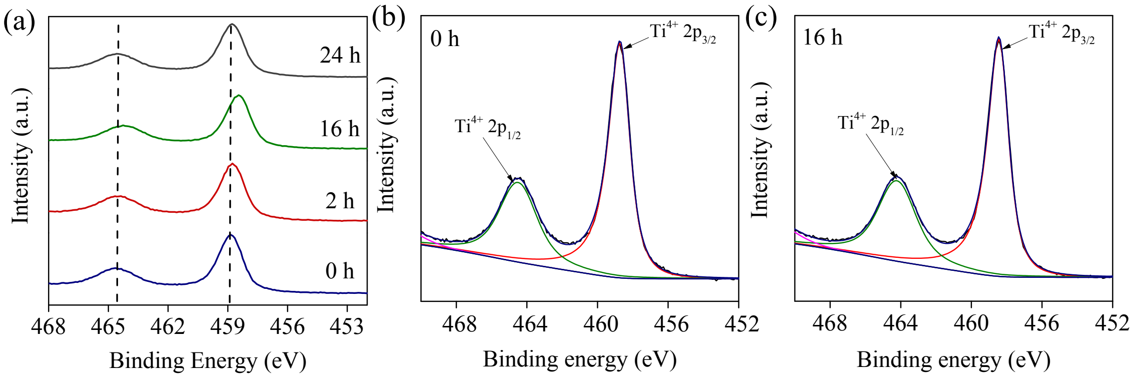


**Figure S3.** (a) High-resolution XPS spectra of Ti 2p at different $NaBH_4$ treatment times. Deconvoluted Ti 2p core level spectra of (b) pure and (c) $TiO_{2-x}$ film treated for 16 h.

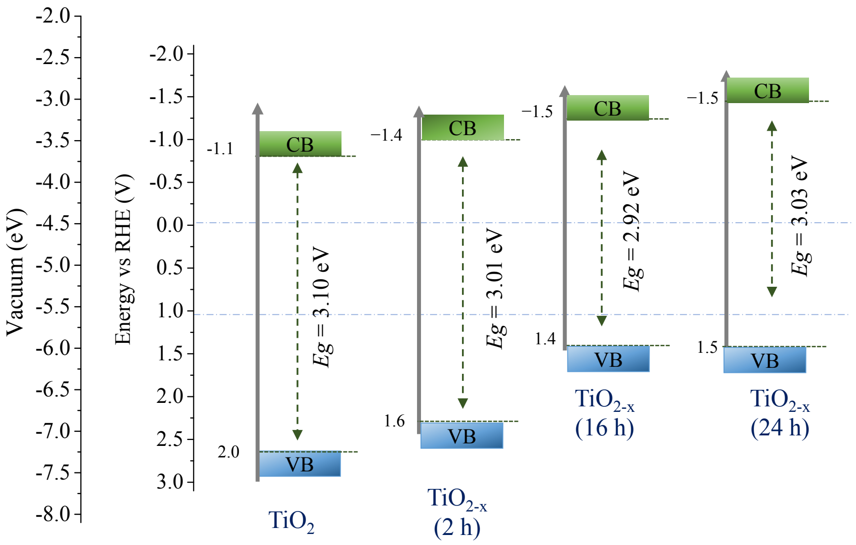


**Figure S4.** The schematic illustration of the band positions for the pristine $TiO_2$ and treated $TiO_{2-x}$ at different reduction times, band gap structure for $TiO_2$ and $TiO_{2-x}$ NTAs were obtained based on the VB position from XPS results and optical band gap from the absorption spectroscopy.

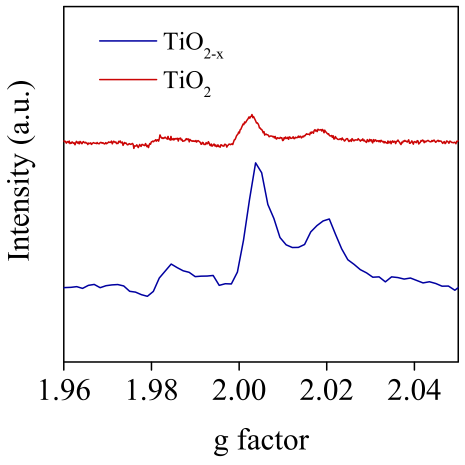


**Figure S5.** EPR spectra for $TiO_2$ and $TiO_{2-x}$ NTAs measured at 50 K.

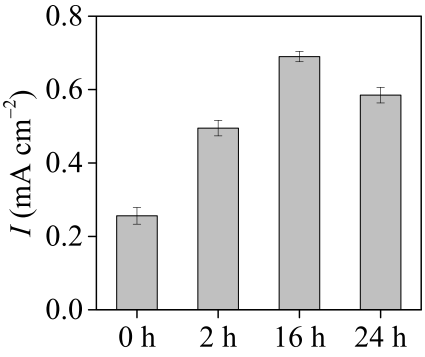


**Figure S6**. Measured photocurrent density of the reduced $TiO_2$ NTAs at 1.7 vs. RHE as a function of $NaBH_4$ treatment time in 0.1M $Na_2SO_4$ (pH 5) recorded at 5 mV/s ($\lambda_{ex}$ =370 nm).

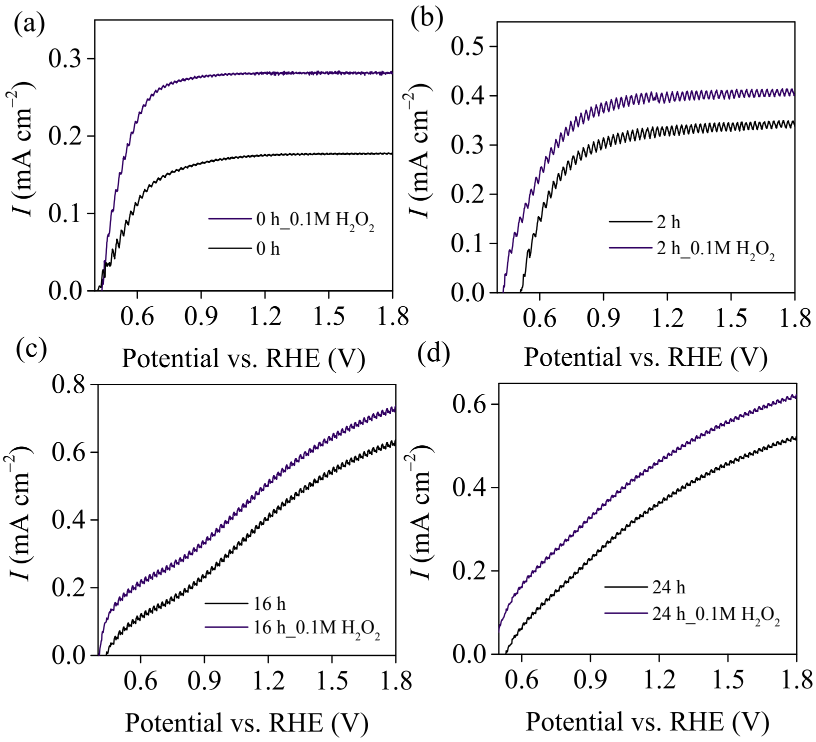


**Figure S7.** LSV in 0.1M $Na_2SO_4$ with and without 0.1M $H_2O_2$ recorded at 5 mV/s under light ($\lambda_{ex}$=370 nm) of (a) $TiO_2$ and $TiO_{2-x}$ NTAs at different reduction times: (b) 2 h, (c) 16 h, and (d) 24 h.

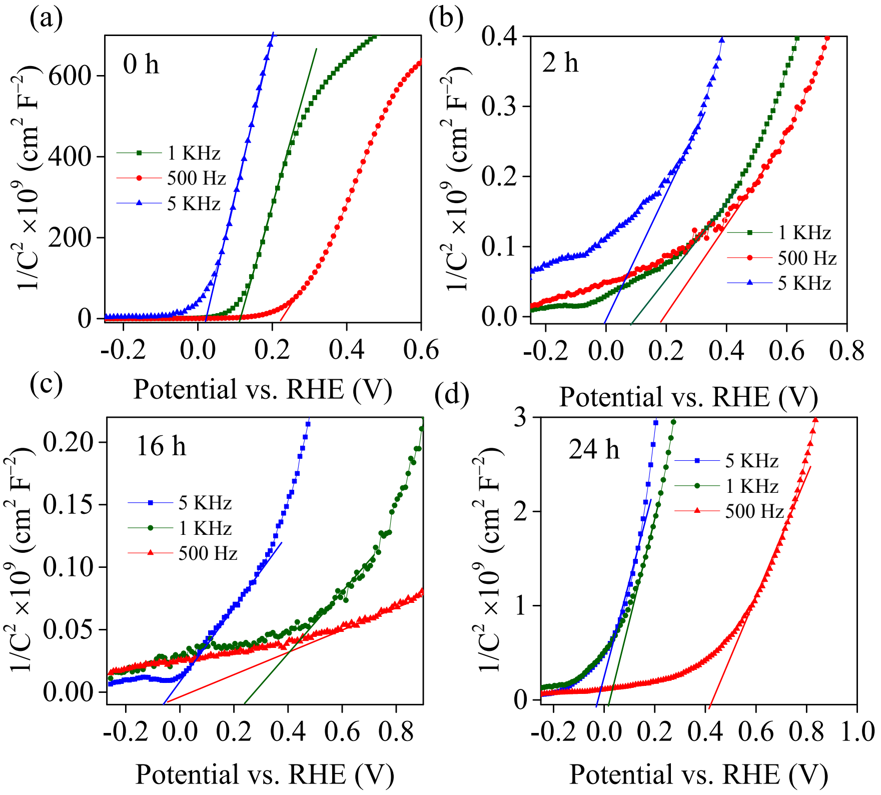


**Figure S8.** Mott–Schottky plots at different frequencies of (a)$TiO_2$ (0 h) and $TiO_{2-x}$ nanotubes obtained by treatment in 1M $NaBH_4$ solution at different times (b) 2 h, (c) 16 h, and (d) 24 h.

**Table S1.** Flat band potential values for selected frequency for $TiO_2$ and $TiO_{2-x}$ samples.

| Sample | $V_{FB}$ (V) for 500 Hz | $V_{FB}$ (V) for 1 KHz | $V_{FB}$ (V) for 5 KHz |
|---|---|---|---|
| **0 h** | 0.22 ± 0.02 | 0.11 ± 0.02 | 0.02 ± 0.02 |
| **2 h** | 0.17 ± 0.02 | 0.14 ± 0.02 | -0.01 ± 0.02 |
| **16 h** | -0.06 ± 0.02 | 0.21 ± 0.02 | -0.08 ± 0.02 |
| **24 h** | 0.40 ± 0.02 | 0.01 ± 0.02 | -0.04 ± 0.02 |

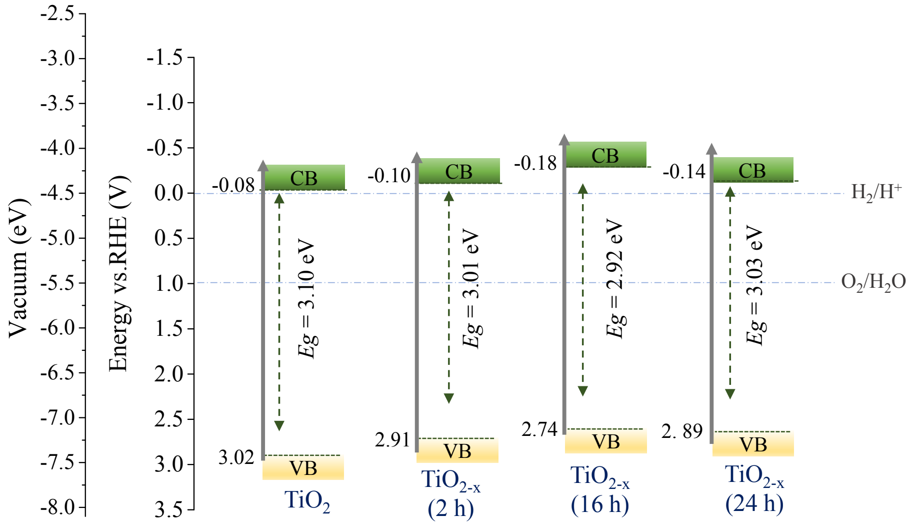


**Figure S9.** The schematic illustration of the band positions for the pristine $TiO_2$ and treated $TiO_{2-x}$ at different reduction times, band gap structure for $TiO_2$ and $TiO_{2-x}$ NTAs were obtained based on the CB position from M-S results and optical band gap from the absorption spectroscopy.

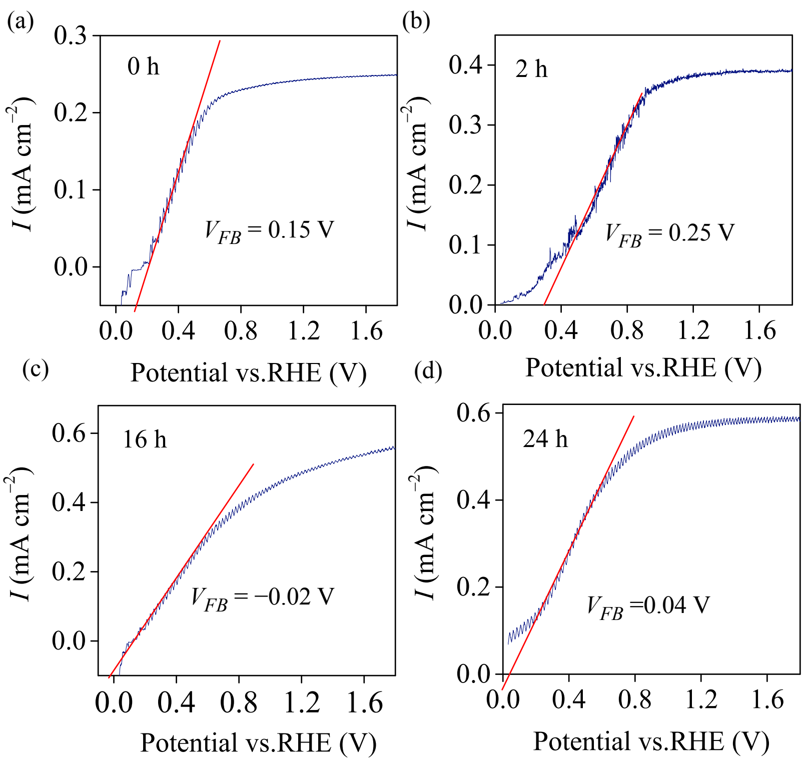


**Figure S10.** LSV in 0.1M $Na_2SO_4$ (pH 5) recorded at 5 mV/s of $TiO_2$ and $TiO_{2-x}$ NTAs at different reduction times (2 h, 16 h, 24 h) under light illumination.

**Table S2.** Summary of PEC properties for $TiO_2$ and $TiO_{2-x}$ samples. The onset potential and flat band potentials were determined using a Gärtner- Butler and Mott–Schottky (5kHz) model.

| Sample | $V_{FB}$ (V) by G-B model | $V_{FB}$ (V) by M-S model |
|---|---|---|
| **0 h** | 0.15 ± 0.02 | 0.02 ± 0.02 |
| **2 h** | 0.25 ± 0.02 | -0.01 ± 0.02 |
| **16 h** | -0.04 ± 0.02 | -0.08 ± 0.02 |
| **24 h** | 0.04 ± 0.02 | -0.04 ± 0.02 |
